# High intensity interval training protects the heart during increased metabolic demand in patients with type 2 diabetes: a randomised controlled trial

**DOI:** 10.1007/s00592-018-1245-5

**Published:** 2018-11-01

**Authors:** Jose Suryanegara, Sophie Cassidy, Vladan Ninkovic, Dejana Popovic, Miljan Grbovic, Nduka Okwose, Michael I. Trenell, Guy G. MacGowan, Djordje G. Jakovljevic

**Affiliations:** 1 Faculty of Medical Sciences, Cardiovascular Research Centre, Institutes of Cellular and Genetic Medicine, Newcastle University, 4th Floor William Leech Building M4.074, Newcastle upon Tyne, NE2 4HH UK; 2 Department of Cardiology, Specialist Hospital for Diabetes Merkur, Vrnjacka Banja, Serbia; 3 Faculty of Medicine and Pharmacy, University of Belgrade, and Cardiology Department, Clinical Centre Serbia, Belgrade, Serbia; 4 Faculty of Sport and Physical Education, University of Belgrade, Belgrade, Serbia; 5 Newcastle upon Tyne Hospitals NHS Foundation Trust, Newcastle upon Tyne, UK; 6 RCUK Newcastle Centre for Ageing and Vitality, Newcastle University, Newcastle upon Tyne, UK

**Keywords:** Cardiac function, Type 2 diabetes, HIIT, Submaximal exercise

## Abstract

**Aim:**

The present study assessed the effect of high intensity interval training on cardiac function during prolonged submaximal exercise in patients with type 2 diabetes.

**Methods:**

Twenty-six patients with type 2 diabetes were randomized to a 12 week of high intensity interval training (3 sessions/week) or standard care control group. All patients underwent prolonged (i.e. 60 min) submaximal cardiopulmonary exercise testing (at 50% of previously assess maximal functional capacity) with non-invasive gas-exchange and haemodynamic measurements including cardiac output and stroke volume before and after the intervention.

**Results:**

At baseline (prior to intervention) there was no significant difference between the intervention and control group in peak exercise oxygen consumption (20.3 ± 6.1 vs. 21.7 ± 5.5 ml/kg/min, *p* = 0.21), and peak exercise heart rate (156.3 ± 15.0 vs. 153.8 ± 12.5 beats/min, *p* = 0.28). During follow-up assessment both groups utilized similar amount of oxygen during prolonged submaximal exercise (15.0 ± 2.4 vs. 15.2 ± 2.2 ml/min/kg, *p* = 0.71). However, cardiac function i.e. cardiac output during submaximal exercise decreased significantly by 21% in exercise group (16.2 ± 2.7–12.8 ± 3.6 L/min, *p* = 0.03), but not in the control group (15.7 ± 4.9–16.3 ± 4.1 L/min, *p* = 0.12). Reduction in exercise cardiac output observed in the exercise group was due to a significant decrease in stroke volume by 13% (*p* = 0.03) and heart rate by 9% (*p* = 0.04).

**Conclusion:**

Following high intensity interval training patients with type 2 diabetes demonstrate reduced cardiac output during prolonged submaximal cardiopulmonary exercise testing. Ability of patients to maintain prolonged increased metabolic demand but with reduced cardiac output suggests cardiac protective role of high intensity interval training in type 2 diabetes.

**Trial registration:**

ISRCTN78698481. Registered 23 January 2013, retrospectively registered.

## Introduction

Diabetes is one of the major risk factors of cardiovascular complications. Patients with type 2 diabetes are at significantly increased risk for cardiovascular morbidity and mortality compared with age-matched healthy control subjects [1, 2]. The high risk of cardiovascular mortality and morbidity in diabetes is associated with alteration in cardiac function and structure [3, 4]. Besides administration of medication and dietary intervention, exercise has been widely known for many years to be the basis of treating patients with type 2 diabetes and preventing further complications as well as reduce cardiovascular morbidity and mortality [5]. However, to obtain most benefits from exercise, intensity, duration and type of exercise should be taken into consideration [6]. Evidence showed that substituting moderate intensity exercise with some vigorous exercise is more effective in terms of reducing high blood glucose and improving cardiorespiratory fitness [7].

High intensity interval training can be characterized by brief period of vigorous exercise intercepted by short periods of rest [8]. Although several studies reported cardiac structural and functional changes following exercise training in patients with type 2 diabetes [3, 8], very few investigated the effect of high intensity interval training on cardiac function and performance in response to stress. It has been suggested that exercise training reduces vascular resistances during submaximal exercise in obese metabolic syndrome individuals [9].

It appears, however, that no study defined the effect of high intensity interval training on cardiac function during prolonged submaximal exercise in patients with type 2 diabetes. This is clinically important because interventions that may demonstrate reduced cardiac work in response to increased metabolic demand may suggest cardiac protective role of such intervention. The aim of the present study was to define the effect of high intensity interval exercise training on cardiac function during prolonged submaximal exercise in patients with type 2 diabetes. We hypothesized that patients with type 2 diabetes will demonstrate reduced cardiac output (i.e. less cardiac work) for the same metabolic demand following high intensity interval training.

## Methods

### Study population

Out of 28 initially screened patients with type 2 diabetes, 26 met study inclusion criteria and were willing to participate in the study. Patients were randomized to a control group and an intervention, i.e. high intensity interval training group. The study inclusion criteria included patients with confirmed diagnosis of type 2 diabetes which was diet and/or metformin controlled for minimum of 6 months prior study, absence of diabetic complications, non-smokers and without limitations to exercise. Participants were excluded from the study if they had a history of coronary artery disease, taking medication known to effect cardiorespiratory function, or were routinely doing regular moderate to vigorous exercise. Participants were also excluded if they had contraindication to cardiopulmonary exercise testing. Ethical approval was obtained from Newcastle and Northeast Tyneside Local Research Ethics Committee and all study procedures were performed in accordance with Declaration of Helsinki. Eligible patients provided written informed consent.

### Equipment and procedure

At the screening visit the following assessments were performed: medical history, physical examination, blood sampling, 12-lead electrocardiography, and blood pressure. Then, patients were allocated into intervention and control group using randomization table. General information such as glycaemic control, lipid profile and body anthropometrics were evaluated at baseline and following intervention. All participants underwent cardiopulmonary exercise testing on three occasions i.e. maximal exercise test was conducted as part of the screening and safety procedure to ensure normal cardiovascular response to high intensity exercise. Baseline (pre-intervention) submaximal exercise took place a week after the maximal exercise test. Follow-up (post-intervention) submaximal exercise was performed 3 days following the final high intensity interval training session.

Maximal and submaximal exercise tests were conducted on an electromagnetically braked recumbent cycle ergometer (Corival, Lode, Groningen Netherland). During maximal exercise test, subjects started to cycle at resistance of 40 W and increased continuously 15 W per min. Maximal oxygen consumption was achieved when subjects reached volitional exhaustion or were unable to continue cycling at speed of 60–70 rev/min. Active support and motivation were continuously given during the test to encourage maximal volitional effort. Meanwhile, a 60-min submaximal exercise test was performed at the intensity of 50% of maximal oxygen consumption. Gas exchange and haemodynamic measurements were performed at rest and during submaximal exercise. Prior to exercise test, gas exchange and haemodynamic measurements were performed during a 20-min resting period.

Participants were advised to abstain from exercise as well as alcohol, tobacco and caffeine for the last 24 h before research visit. Expired gases were assessed using the face mask and online metabolic gas exchange system (Cortex metalyser 3B, Lepizig, Germany).

Central haemeodynamics measures including cardiac output, stroke volume and heart rate were measured at rest and during submaximal exercise using a validated non-invasive continuous bioreactance method (NICOM, Cheetah Medical, Delaware, USA) [10, 11]. The bioreactance evaluates changes of an oscillating current voltage traversing the thoracic cavity. The bioreactance system consists of four dual surface electrode and a radio frequency generator that radiates high frequency current across the thorax. The first two electrodes were put on the left side while the other two were placed on the right side of the thorax. The bioreactance assesses the relative phase shift of current across the thorax and estimates cardiac output based on blood flow through aorta as previously detailed [10]. Arteriovenous oxygen difference was calculated as the ratio between O_2_ consumption and cardiac output, and represents the ability of the skeletal muscles to extract delivered O_2_.

### Intervention

High intensity interval training group performed three times per week for 12 weeks training with 36-cycle ergometry sessions at a gym. Borg Rating of Perceived Exertion was used to determine exercise intensity, with scale ranging from 6 to 20 [12]. Every exercise intervention started with 5 min of warm up with the purpose of increasing intensity from 9 (very light) to 13 (somewhat hard). Then it progressed to intensity 16–17 (very hard) with pedal rate > 80 rev/min for five intervals. In the first week, each interval lasted for 2 min and it inclined 10 s for every week until it reached 3 min and 50 s of interval after 12 weeks of training. Each interval was followed with 3 min recovery cycle including 90 s of passive recovery [3]. The first session of exercise training was supervised while the remaining sessions were conducted using guidance from an iPod (Apple, CA, USA) through voice-recorded instruction. To assess exercise adherence, an exercise diary and weekly phone call were used. Adequate adherence was strictly determined by minimum of 32 out of a possible 36 sessions from the diary. In addition, during the 12 weeks of exercise intervention, participants may continue their normal habits and were not asked to alter their medication, diet and routine physical activity [3].

### Sample size and statistical analysis

It was calculated that a sample size of 24 patients (12 per group) will be sufficient to detect a clinically meaningful difference (change) in submaximal exercise cardiac output in response to intervention of 1.8 L/min, providing the study power of 80%, a two-sided α of 5%, and allowing for 10% withdrawals.

Statistical analysis was conducted using IBM SPSS Statistics software (version 22, NY, USA). Normality of distribution was checked using histogram and normal probability plots. Further normality was tested using Shapiro–Wilk test. Independent samples *t* test was used for comparing baseline variables. Comparison between continuous variables was assessed with paired *t* test, and ANCOVA was used for between groups’ comparisons with baseline value as covariate. For non-normally distributed data, Wilcoxon signed-rank test was performed as a substitute non parametric test. An alpha level of 0.05 was set and *p* < 0.05 were considered statistically significant. All data are displayed as mean ± SD unless indicated otherwise.

## Results

The study included 13 subjects in exercise group (3 men, 10 women) and 13 subjects in control groups (3 men, 10 women). There were no significant differences in demographic and baseline characteristics between high intensity interval training and control groups (Table 1).

**Table 1:** Baseline characteristic of study participants

	Control	Exercise	*p* value
Age (years)	59.8 ± 8.6	61.1 ± 8.6	0.73
Gender (men/women)	3/10	3/10	N/A
Height (cm)	169.8 ± 8.6	170.4 ± 7.6	0.85
Weight (kg)	91.0 ± 9.8	90.5 ± 15.0	0.91
Duration of diabetes (years)	4.3 ± 1.4	4.8 ± 1.2	0.64
Body mass index (kg/m^2^)	31.9 ± 5.3	31.3 ± 5.4	0.77
Body surface area (m^2^)	2.03 ± 0.1	2.04 ± 0.2	0.92
Systolic blood pressure (mmHg)	141.4 ± 13.6	142.3 ± 17.3	0.89
Diastolic blood pressure (mmHg)	86.1 ± 10.4	89.0 ± 12.7	0.52
Fasting glucose (mmol/L)	6.8 ± 0.8	6.6 ± 1.6	0.67
HbA_1c_ mmol/mol	55.5 ± 6.0	53.6 ± 10.5	0.59
Triglyceride mmol/L	1.1 ± 0.4	1.1 ± 0.3	0.92
Total cholesterol mmol/L	4.33 ± 1.0	4.18 ± 1.1	0.74
Alanine aminotransferase (units/L)	37.2 ± 18.4	34.5 ± 11.5	0.67

At the baseline, there were no significant difference between the groups in fasting glucose (exercise, 6.6 ± 1.6 mmol/L vs. control, 6.8 ± 0.8 mmol/L, *p* = 0.67) as well as glycated hemoglobin (exercise, 53.6 ± 10.5 mmol/mol vs. control, 55.5 ± 6.0 mmol/mol, *p* = 0.59). At the follow-up assessment there was a significant increase in fasting glucose in the control group by 1.8 mmol/L (to 7.6 ± 1.4 mmol/L, *p* = 0.03) while the fasting glucose for exercise group remained unchanged (6.6 ± 1.6–6.8 ± 1.7 mmol/L, *p* = 0.15). There were no significant changes in glycated hemoglobin and body weight following the 12-week period in either group.

Maximal cardiopulmonary exercise testing was conducted at the screening visit to determine functional capacity and cardiorespiratory fitness. There were no significant differences between gas exchange and haemodynamic variables at rest (Table 2) including heart rate, oxygen consumption and blood pressure. At maximal exercise, control group demonstrated significantly higher systolic blood pressure compared to exercise group (*p* = 0.05, Table 2), whereas other physiological measurements including arteriovenous O_2_ difference were not significantly different between the two groups. These data indicate that before exercise training, both exercise and control groups had the same level of cardiorespiratory fitness.

**Table 2:** Resting and peak exercise gas exchange and heamodynamic measurements

	Control	Exercise	*p* value
Resting measurements			
Heart rate (beats/min)	79.3 ± 11.0	76.3 ± 11.2	0.27
Oxygen consumption (ml/kg/min)	3.2 ± 0.6	3.3 ± 1.4	0.42
Oxygen consumption (l/min)	0.3 ± 0.1	0.3 ± 0.1	0.48
Ventilation (L/min)	10.3 ± 2.8	10.5 ± 3.3	0.44
Respiratory exchange ratio	0.9 ± 0.1	0.9 ± 0.2	0.28
Systolic blood pressure (mmHg)	142.1 ± 14	142.4 ± 18.3	0.48
Diastolic blood pressure (mmHg)	85.2 ± 10.5	87.4 ± 12.7	0.33
Metabolic equivalent (ml/kg/min)	0.9 ± 0.2	0.9 ± 0.4	0.39
Peak exercise measurements			
Heart rate (beats/min)	156.3 ± 15.0	153.8 ± 12.5	0.28
Oxygen consumption (ml/kg/min)	20.3 ± 6.1	21.7 ± 5.5	0.21
Oxygen consumption (l/min)	1.9 ± 0.3	2.0 ± 0.6	0.17
Ventilation (L/min)	71.2 ± 12.1	71.6 ± 17.6	0.48
Respiratory exchange ratio	1.2 ± 0.1	1.2 ± 0.1	0.45
Systolic blood pressure, mmHg)	212.6 ± 11.0	179.1 ± 62.7	0.05
Diastolic blood pressure (mmHg)	107.9 ± 20.0	100 ± 16.3	0.16
Metabolic equivalent (ml/kg/min)	5.7 ± 1.8	6.4 ± 1.5	0.18
Work rate (watts)	129 ± 30	135 ± 34	0.31

**Fig. 1:**
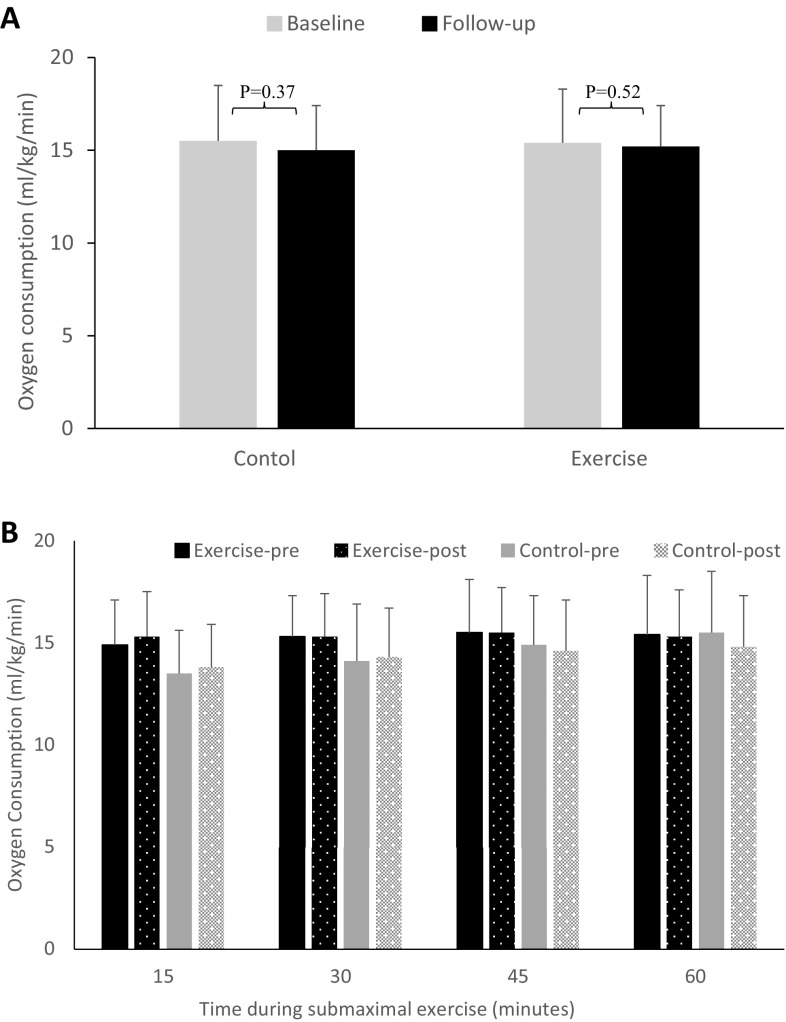
Effect of high intensity interval training on oxygen consumption during prolonged submaximal exercise testing

During follow-up visit, cardiac function and performance were assessed at rest and during prolonged submaximal exercise testing. Gas exchange and hemodynamic variables before and after 12 weeks in control and exercise group measured at rest and during prolonged submaximal exercise test are presented in Tables 3 and 4. Oxygen consumption during submaximal exercise, as the measure of cardiorespiratory fitness, did not change in response to intervention (*p* = 0.71 between the groups, Table 4), with both groups showing similar level of oxygen utilisation in response to submaximal exercise following the 12-week period (Fig. 1). These findings clearly suggest that high intensity interval training had no effect on level of oxygen consumption during prolonged submaximal work in patients with type 2 diabetes.

**Table 3:** The effect of high intensity interval training on metabolism and cardiac function at rest

Physiological variables at rest	Control			Exercise			*p* value between groups
	Pre	Post	*p* value	Pre	Post	*p* value	
Oxygen consumption (L/min)	0.3 ± 0.1	0.3 ± 0.0	0.18	0.3 ± 0.1	0.3 ± 0.1	0.62	0.97
Oxygen consumption (ml/min/kg)	2.8 ± 0.7	2.9 ± 0.4	0.22	2.8 ± 0.5	3.0 ± 0.3	0.17	0.57
Carbon dioxide production (L/min)	0.2 ± 0.1	0.2 ± 0.0	0.39	0.22 ± 0.1	0.2 ± 0.1	0.28	0.91
Respiratory exchange ratio	0.9 ± 0.1	0.9 ± 0.1	0.24	0.8 ± 0.1	0.8 ± 0.1	0.31	0.83
Stroke volume (ml/beat)	82.4 ± 15.0	81.4 ± 15.6	0.45	78.4 ± 14.8	83.3 ± 12.1	0.34	0.21
Heart rate (beats/min)	78.4 ± 19.0	76.6 ± 7.8	0.13	77.9 ± 10.1	72.1 ± 10.0	0.06	0.18
Cardiac output (L/min)	6.5 ± 2.6	6.2 ± 1.0	0.23	6.1 ± 1.2	6.0 ± 6.0.8	0.15	0.58
Arteriovenous oxygen difference (mL/100 mL)	4.6 ± 1.2	4.8 ± 1.1	0.64	4.9 ± 1.0	5.0 ± 1.2	0.71	0.42

**Table 4:** The effect of high intensity interval training on metabolism and cardiac function during submaximal exercise

Physiological Variables during Submaximal Exercise	Control			Exercise			*p* value between groups
	Pre	Post	*p* value	Pre	Post	*p* value	
Oxygen consumption (L/min)	1.4 ± 0.2	1.35 ± 0.17	0.08	1.4 ± 0.4	1.4 ± 0.3	0.34	0.55
Oxygen consumption (ml/min/kg)	15.5 ± 3.1	15.0 ± 2.4	0.37	15.4 ± 2.9	15.2 ± 2.2	0.52	0.71
Carbon dioxide production (L/min)	1.3 ± 0.2	1.97 ± 0.17	0.12	1.4 ± 0.4	1.3 ± 0.3	0.27	0.79
Respiratory exchange ratio)	0.96 ± 0.04	0.96 ± 0.06	0.5	1.0 ± 0.1	1.0 ± 0.1	0.41	0.62
Stroke volume (ml/beat)	127.1 ± 25.5	124.7 ± 32.6	0.12	128.1 ± 21.2	110.9 ± 22.1	0.03	0.04
Heart rate (beats/min)	122.7 ± 10.4	126.5 ± 15.5	0.18	125.4 ± 16.3	114.4 ± 13.2	0.04	0.05
Cardiac output (L/min)	15.7 ± 4.9	16.3 ± 4.1	0.12	16.2 ± 2.7	12.8 ± 3.6	0.03	0.05
Arteriovenous oxygen difference (mL/100 mL)	8.9 ± 2.2	8.3 ± 2.6	0.21	8.6 ± 2.5	10.9 ± 3.2	0.03	0.04
Work rate (watts)	63 ± 16	64 ± 15	0.44	67 ± 14	65 ± 16	0.31	0.18

Resting haemodynamic measurements such as cardiac output, stroke volume, heart rate, and arterial blood pressure were not significantly different before and after the 12-week period in both intervention and control group, not either there was significant change between the groups (Table 3).

**Fig. 2:**
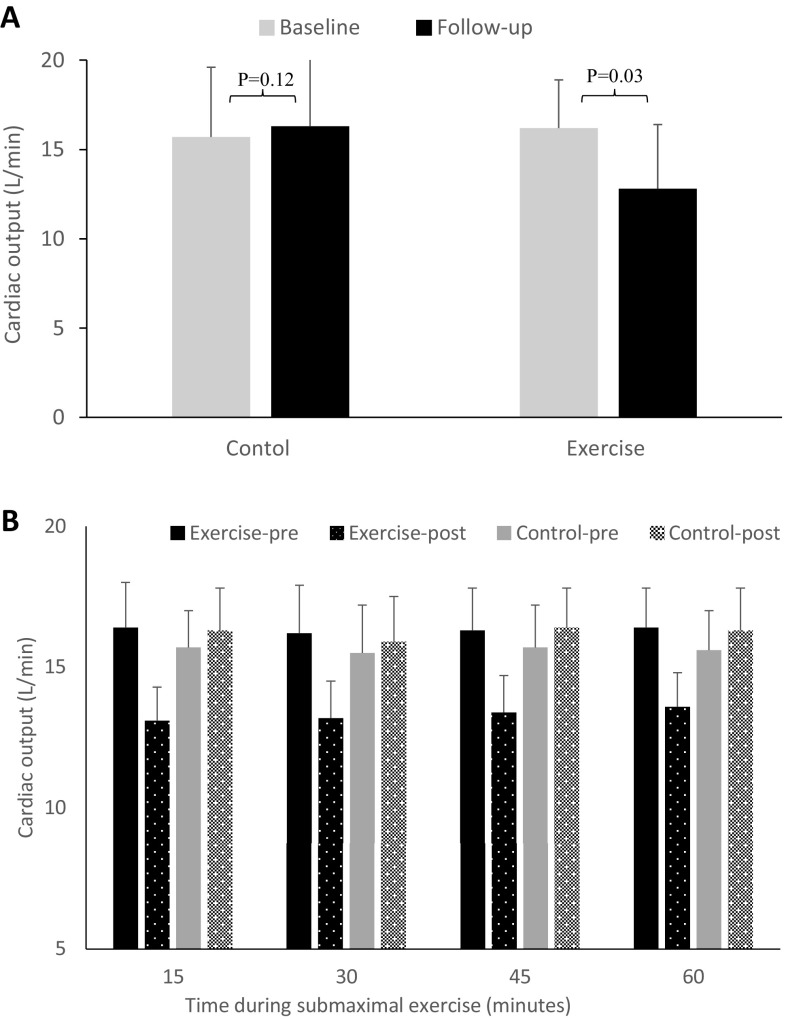
Effect of high intensity interval training on cardiac output during prolonged submaximal exercise testing

Submaximal haemodynamic measurements were significantly different before and after the 12-week period between the groups. Cardiac function during prolonged submaximal exercise, represented by cardiac output, decreased significantly by 21% in exercise group (16.2 ± 2.7–12.8 ± 3.6 L/min, *p* = 0.03), but not in the control group (15.7 ± 4.9–16.3 ± 4.1 L/min, *p* = 0.12, Table 3; Fig. 2). Reduction in exercise cardiac output observed in the intervention group was due to a significant decrease in stroke volume by 13% (*p* = 0.03) and heart rate by 9% (*p* = 0.04). Reduced cardiac response to prolonged submaximal exercise testing with sustained metabolic demand suggests cardiac protective role of high intensity interval training.

Results further demonstrate a significant 21% increase in peak exercise arterial-venous oxygen difference (Table 4). This suggests that improved oxygen extraction is the main adaptive mechanisms to explain ability to maintained submaximal oxygen consumption with reduced cardiac output.

## Discussion

The present study is the first to assess the effect of high intensity interval training on cardiac output response to prolonged submaximal exercise in patients with type 2 diabetes. The two major findings suggest that high intensity interval training (i) did not alter metabolic and haemodynamic function at rest, and (ii) was associated with reduced cardiac output but improved oxygen extraction during prolonged submaximal effort suggesting protective cardiac role.

The purpose of treatment in type 2 diabetes is to maintain the blood glucose, lipid as well as blood pressure level in control to prevent further complications [13]. Exercise has been recognized as an effective method in diabetic management [5]. The vast majority of the studies mainly explored the association between exercise and prevention of cardiac complications in type 2 diabetes [2]. Although study about the effect of high intensity interval training has been conducted previously [8], no study investigated its effect on heart function during prolonged submaximal exercise in patients with type 2 diabetes. This is an important study because people in their everyday life are likely to engage with physical activity that are of low to submaximal rather than high intensity effort. Therefore, identifying strategies to protect the heart during the stress may play a major role in prevention of cardiovascular complications in type 2 diabetes. It appears that high intensity interval training was an effective method to reduce cardiac stress during prolonged metabolic demand.

High intensity exercise training did not affect fasting glucose level. This data are in line with previous study which also suggested no change fasting glucose following intervention [14]. Similar results are also shown by Dunstan et al. as they demonstrated unchanged fasting glucose and fasting plasma insulin level after 6 months of high intensity interval training in older patients [15]. On another note, it has been suggested that high intensity interval training reduces the post prandial glucose due to improved sensitivity of the GLUT 4 receptor [14, 16]. In addition, it has also been suggested that high intensity interval training may improve insulin sensitivity [17]. Furthermore, it should be noted that in individuals with diabetes high intensity interval training may improve insulin-stimulated glucose uptake at the tissue and organ level as previously suggested [18]. In the present study both exercise and control groups showed no significant change on glycated hemoglobin and body weight following the 12-week period. However, it was previously suggested that improvement in glycated hemoglobin in response to exercise intervention is associated with reduction in body weight [3]. In the present study, there was no change in body weight, therefore, it should not be surprising that there was no change in glycated hemoglobin.

Due to increased sympathetic activity, it was suggested that patients with type 2 diabetes commonly demonstrate higher resting heart rate compared to healthy individuals exposing them to increased risk of cardiovascular morbidity and mortality [19]. Mechanism of sympathetic activation and increased resting heart rate include metabolic syndrome, abdominal obesity and insulin resistance [19]. In the present study, patients in both groups demonstrate resting heart rate which does not appear to be elevated and certainly not reaching threshold of tachycardia despite being obese with body mass index > 30 kg/m^2^. It is, therefore, not surprising that high intensity interval training did not induce reduction in resting heart rate. However, following the 12-week period there was a significant decline in submaximal heart rate in the exercise but not in the control group. This finding is in agreement with previous studies reporting lower heart rate in response to exercise in patients with type 2 diabetes [20–22]. Exercise-induce decline in heart rate is mediated via improved autonomic regulation of the heart i.e. higher parasympathetic or lower sympathetic activity as well as enhanced venous return [23]. Heart rate response to exercise may also be confounded by several factors including age [24]. In the present study the mean age of subjects was ~ 60 years, and it was not significantly different between the groups suggesting for the first time that high intensity interval training induces reduction in heart rate during prolonged submaximal exercise in older patients with type 2 diabetes.

Stroke volume refers to amount of blood ejected from left ventricle per beat. Patients with type 2 diabetes appear to demonstrate blunted stroke volume in response to exercise compared with normal subjects [25, 26]. The decline of stroke volumes is associated with reduced end diastolic volume indicating lower left ventricular filling and decreased systolic reserve [26]. Accumulation of glycation end products in prolonged hyperglycaemia induce fibrosis and reduction of connective tissue flexibility, further leading to an increase in myocardial stiffness [25, 27]. However, in the present study both exercise and control group increased stroke volume from rest to submaximal exercise by ~ 30–40%. Increase in stroke volume is caused by increased venous return and enhanced Frank Starling mechanism as well further leading to increased myocardial contractility [27].

Despite none significant change in resting stroke volume, there was, however, a significant reduction in submaximal stroke volume following high intensity interval training. Previous studies suggested an increase in exercise stroke volume following exercise intervention in healthy individuals and patients with metabolic syndrome [9, 28]. However, it should be noted that the present study aimed to evaluate the effect of high intensity interval training on cardiac response to prolonged submaximal intensity which is more reflection of activities of daily living. Data from the present study are partially supported from Fritzsche et al. who argued that stroke volume decreases during prolonged compared to short duration exercise [27]. However, in the present study the same intensity submaximal exercise test was repeated before and after 12-weeks and it is clear that reduction in stroke volume is a physiological adaptation to high intensity interval training. This finding can potentially be explained by mechanisms involving thermoregulation and redistribution of blood to cutaneous vessels and plasma loss which may lead to a decrease venous return and, therefore, reduction of stroke volume [29].

Cardiac output which reflects cardiac work is the amount of blood pumped by the left ventricle per unit of time and is calculated as the product of stroke volume and heart rate [30]. Considering that both submaximal heart rate and stroke volume declined following high intensity interval training, decline in cardiac output is also expected. These findings are different from those of Hagberg et al. who found increase in submaximal cardiac output in patients with coronary artery disease after 12 months of exercise intervention due to an increase in stroke volume [31]. Patients with coronary artery disease may present with impaired Frank-Starling mechanism, and exercise intervention in such circumstances may lead to improvements in haemodynamic response to stress. Similarly, Mora-Rodriguez and colleagues demonstrated an increase in cardiac output following exercise intervention on obese individuals with metabolic syndrome [9]. However, it should be noted that duration of submaximal exercise as well as methodology employed to evaluate haemodynamic function in response to exercise were different between previous and present study i.e. duration and intensity of exercise (20 vs. 60 min), and single rebreathing vs. continuous beat-by-beat stroke volume and cardiac output measurements. These differences in study design as well as patient groups are likely to explain differences in major findings of the present and previous studies [9, 31].

Considering that patients with type 2 diabetes in the present study managed to increase their cardiac output in response to submaximal exercise testing before the intervention suggests that their cardiac function has not being impaired. Therefore, based on a significant reduction in submaximal cardiac output following intervention, it is reasonable to suggest that high intensity interval training may provide cardiac protective role in patients with type 2 diabetes during prolonged and increased metabolic demand.

Additionally, along with a significant reduction in cardiac output it should be noted that patients in the intervention group demonstrated a significant increase in arteriovenous O_2_ difference (i.e. ability of skeletal muscle to extract delivered O_2_). Oxygen consumption (which is the product of cardiac output and arteriovenous O_2_ difference) remained unchanged during prolonged submaximal exercise after high intensity interval training despite reduced cardiac output. These findings indicate that improved O_2_ extraction is the mechanism to explain physiological adaptation to high intensity interval training and ability to sustain oxygen consumption during prolonged submaximal exercise despite reduced cardiac work. This notion has been previously suggested by Heinonen and colleagues who highlighted interaction between the central haemodynamics (blood flow and delivery) with peripheral muscle function and O_2_ extraction [32].

### Study limitations

This present study is not without limitations. Firstly, the sample size was rather small to moderate but *a priori* power calculation revealed the minimal number of participants required to detect significant difference in submaximal exercise cardiac output. Second, better marker of cardiac work during submaximal exercise would be cardiac power output, the product of cardiac output and mean arterial blood pressure. However, blood pressure measurements were not recorded during submaximal exercise. Third, study participants were Caucasian, 70% were women, and mainly elderly raising questions about generalizability of findings. Finally, the Borg scale was used to assess exercise intensity. Heart rate monitors are more accurate, however, the scale is a useful and more practical tool to guide exercise intensity in daily practice as heart rate measurement during intervals of high intensity exercise may appear to be challenging to patients [9].

## Conclusion

The major findings of the present study suggest that high intensity interval training was associated with decreased cardiac output during prolonged submaximal exercise. This may suggest cardiac protective role of high intensity interval training in patients with type 2 diabetes i.e. patients are able to maintain prolonged increased metabolic demand with reduced cardiac stress. This may imply that clinical care teams should consider high intensity interval training as an efficient non-pharmacological strategy that may prevent cardiovascular complications in patients with type 2 diabetes.
